# Supplementary material for: Reverberant 3D optical coherence elastography maps the elasticity of individual corneal layers
Source: Nat Commun. 2019 Oct 25;10:4895. doi: 10.1038/s41467-019-12803-4 (PMC6814807; doi:10.1038/s41467-019-12803-4)
Supplement: Supplementary file 1 — Supplementary Information [file 41467_2019_12803_MOESM1_ESM.pdf]

Supplementary information for "Reverberant 3D Optical Coherence Elastography maps the elasticity of individual layers in cornea" by Zvietcovich *et al.*

Supplementary Figure 1

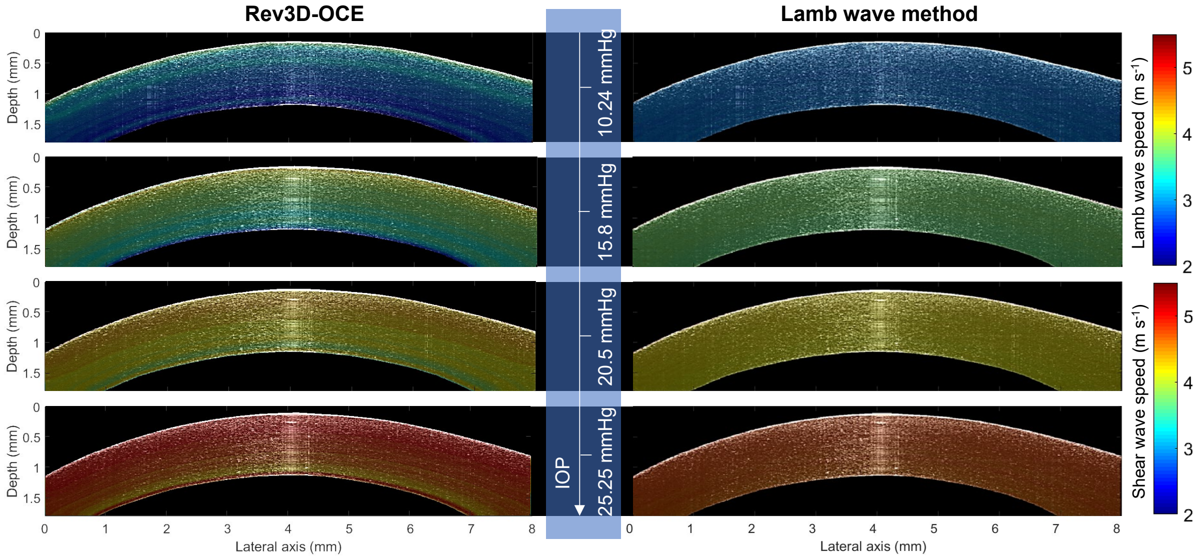

Supplementary Figure 1. Comparison of 2D average depth-dependent shear wave speed superimposed with B-mode structural images provided by Rev3D-OCE (**left column**) and the Lamb wave method (**right column**) of *ex vivo* porcine cornea subjected to four IOP levels: 10.24, 15.80, 20.50, and 25.25 mmHg. All IOP cases have the same shear and Lamb wave speed scale bars valid for the Rev3D-OCE and Lamb wave method, respectively. Rev3D-OCE shear wave speed maps were calculated for a $f_0 = 2$ kHz excitation frequency. Lamb wave speed maps were calculated at a frequency $f_0 = 800$ Hz to cover for all four IOP cases.

Supplementary Figure 2

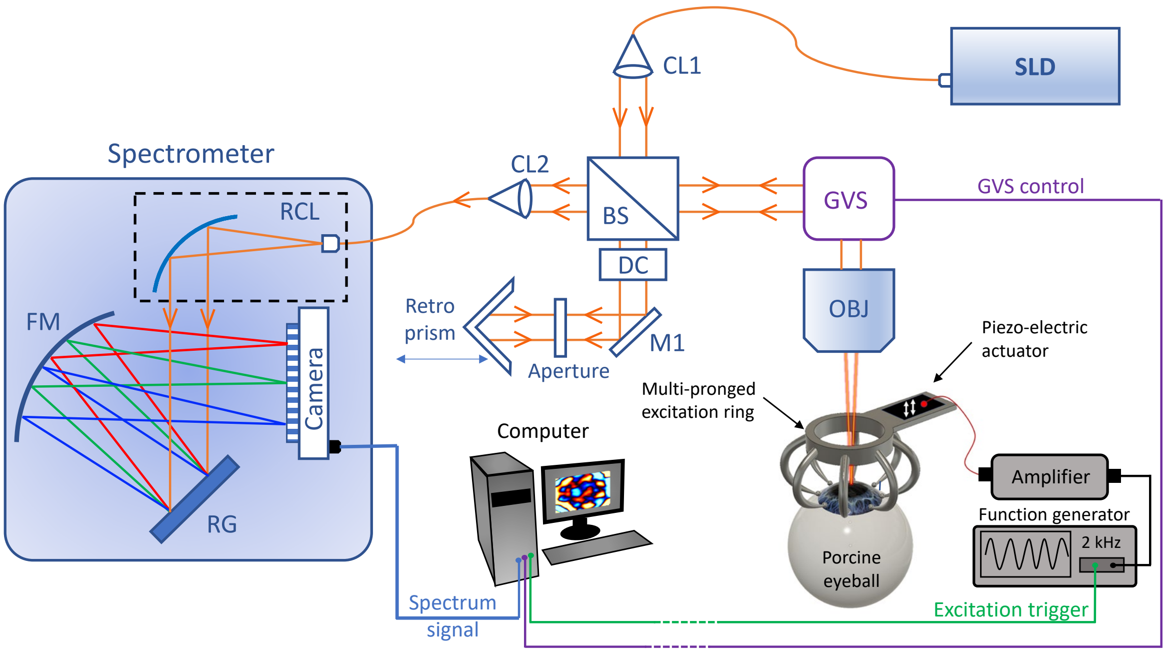

Supplementary Figure 2. Optical and mechanical setup of the experiment. The optical layout consists on a custom built Spectral-domain Phase-sensitive Optical Coherence Tomography (PhS-OCT) system. SLD= superluminescent light emitting diode, CL=collimator, BS=beamsplitter, DC=dispersion compensator, M=mirror, GVS=galvanometric scanner, and OBJ=objective lens. The spectrometer consisted of a reflective collimator (RCL), a reflective grating (RG), a focusing mirror (FM), and a high-speed line-scan sensor (camera). The control of the GVS and acquisition of OCT signals is conducted by a computer. The output OCT beam is going through the objective lens toward the sample (porcine cornea). The cornea is excited by a piezo-electric actuator attached to an eight-head ring. The excitation signal is produced by a function generator and amplified before feeding the transducer. The excitation is triggered by the computer that controls the OCT acquisition and the GVS.

Supplementary Figure 3

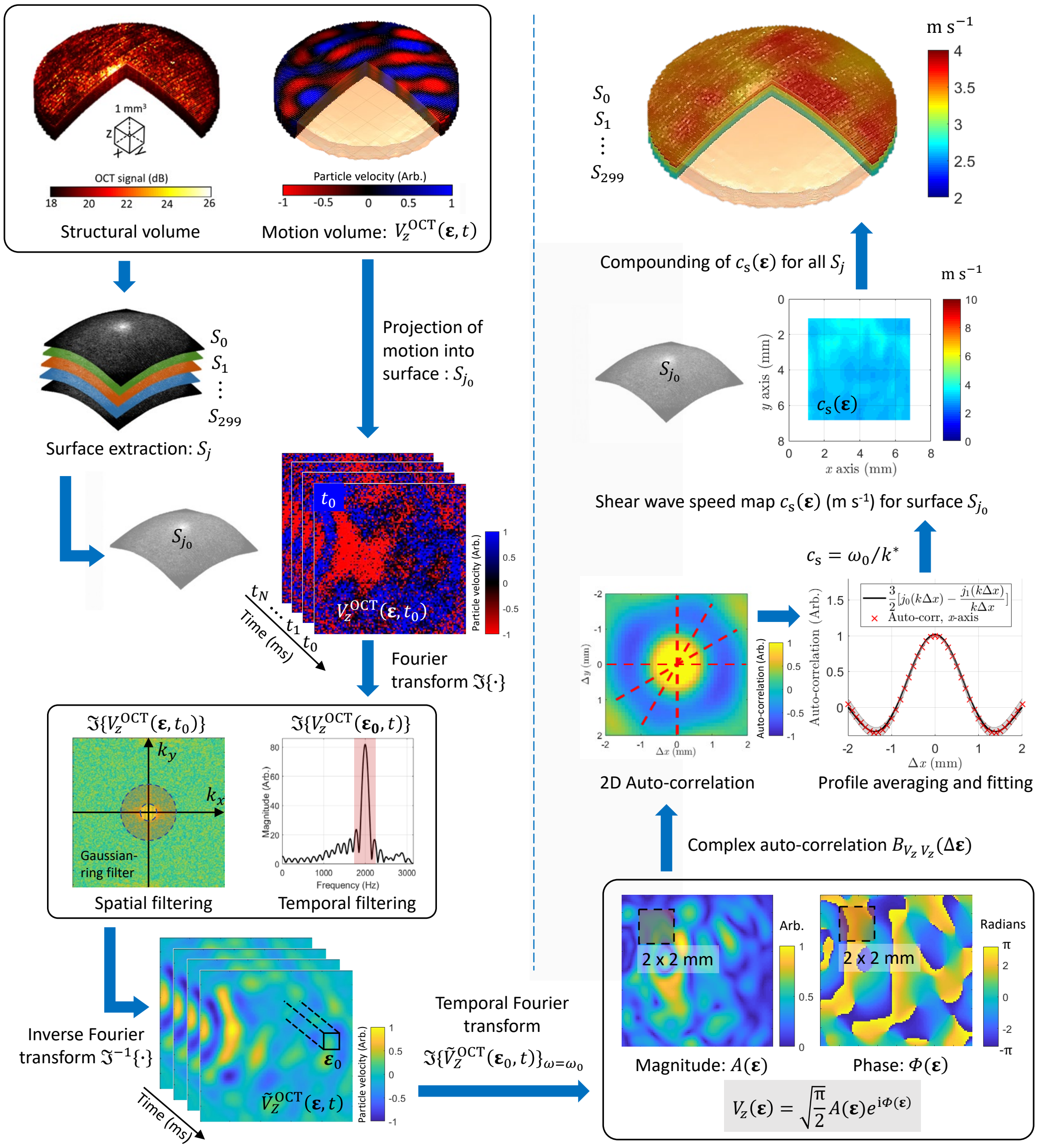

Supplementary Figure 3. Processing pipeline for the Rev3D-OCE method. Structural and motion volumes provided by the PhS-OCT system are used for detecting topographic surfaces (S_j, for $j = 0, 1, 2, \ldots, 299$) in cornea and projections of particle velocity $V_z^{\mathrm{OCT}}(\boldsymbol{\varepsilon}, t)$ on them. Then, temporal and spatial filtering is applied to each projection frame $\tilde{V}_z^{\mathrm{OCT}}(\boldsymbol{\varepsilon}, t)$. A temporal Fourier transform is applied to each spatial location $\boldsymbol{\varepsilon}$ for the calculation of magnitude $A(\boldsymbol{\varepsilon})$ and phase $\Phi(\boldsymbol{\varepsilon})$. Subsequently the complex matrix $V_z(\boldsymbol{\varepsilon}) = \sqrt{\pi/2}\,A(\boldsymbol{\varepsilon})e^{i\Phi(\boldsymbol{\varepsilon})}$ is formed for the local calculation of auto-correlation in a 2 × 2 mm^2 window. The average auto-correlation profile is fitted to Equation 2 of the main manuscript for the local estimation of wave number k^*. Shear wave speed maps $c_s(\boldsymbol{\varepsilon}) = \omega_0/k(\boldsymbol{\varepsilon})^*$ are calculated for each surface S_j in cornea by moving the window through the entire projection frame, and by fixing the *a priori* known excitation frequency $\omega_0 = 2\pi f_0$, where $f_0 = 2$ kHz used during experiments. Finally, the compounding of $c_s(\boldsymbol{\varepsilon})$ calculated at each surface S_j is represented in a speed color-coded 3D volume.

Supplementary Figure 4

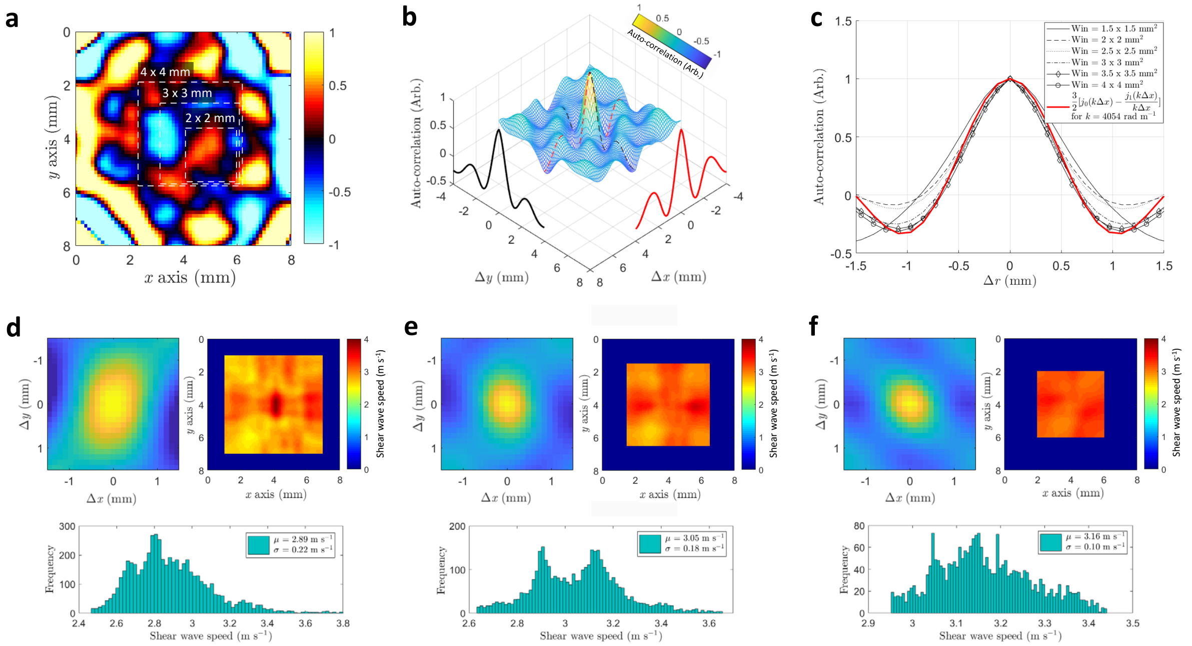

Supplementary Figure 4. Exploration of the impact of the auto-correlation window size in the shear wave speed estimation process using Rev3D-OCE. **(a)** Motion frame representing a 2 kHz reverberant field extracted at the depth $z_0 = 0.30$ mm of a porcine cornea sample. Color bar represents particle velocity in arbitrary units. **(b)** 2D auto-correlation plot of the frame in **(a)** using a 4×4 mm^2 window. Profiles extracted along Δx and Δy are similar but not identical, due to anisotropic properties of the cornea. Colorbar represents normalized auto-correlation in arbitrary units. **(c)** Average of auto-correlation curves ($N = 360$ curves) taken along radial cuts of **(b)** covering 360 degrees when using 6 different window sizes. Average auto-correlation curves tend to approximate the theoretical curve when the window size is increased. **(d-f)** 2D auto-correlation (color map is the same as in **(b)**) plots (**top-left**), 2D shear wave speed (color map is in m s^{-1}) maps (**top-right**), and shear speed distributions (**bottom**) are shown when auto-correlation window sizes of 2×2 mm^2 **(d)**, 3×3 mm^2 **(e)**, and **(f)** 4×4 mm^2 are used in **(a)**. Mean $\pm$ SE are shown in each speed distribution. White discontinuous squares in **(a)** show the auto-correlation window sizes used **in (d-f)**.

Supplementary Figure 5

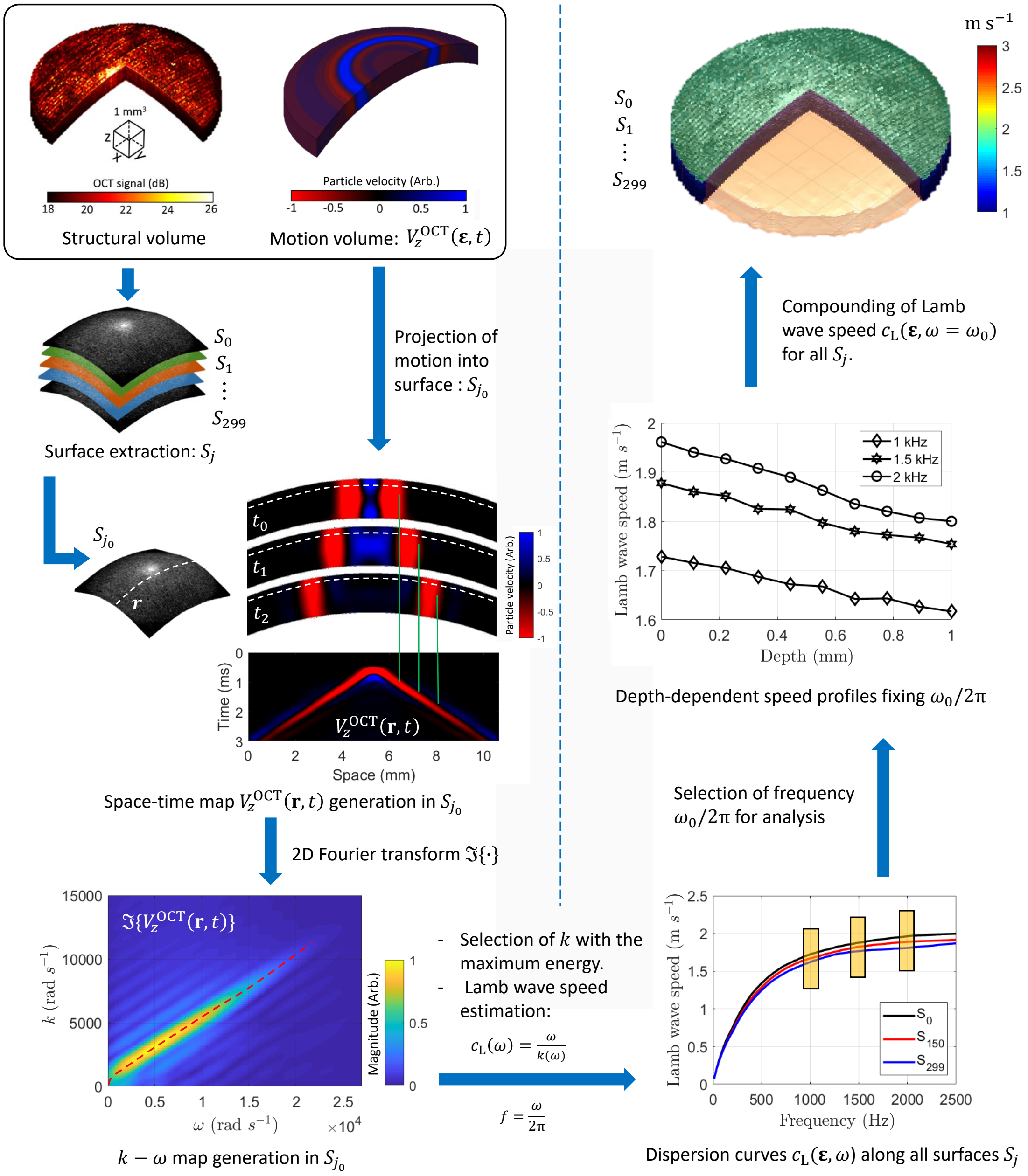

Supplementary Figure 5. Processing pipeline for the Lamb wave method. Structural and motion volumes provided by the PhS-OCT system are used for detecting topographic surfaces (S_j, for $j = 0, 1, 2, \dots, 299$) in cornea, defining wave propagation directions $\hat{\mathbf{r}}$, and projections of particle velocity $V_z^{OCT}(\boldsymbol{\varepsilon}, t)$ on them. Then, for any surface S_j, 2D space-time maps are generated from $V_z^{OCT}(\boldsymbol{\varepsilon}, t)$ along the $\hat{\mathbf{r}}$-t space. Every space-time map is converted into the k-ω space using 2D Fourier transform for a subsequent Lamb wave speed dispersion calculation using $c_L(\omega) = \omega/k^*(\omega)$. Depth-dependent Lamb wave speed plots can be extracted by choosing an excitation frequency $\omega_0 = 2\pi f_0$. Finally, the compounding of $c_L(\boldsymbol{\varepsilon}, \omega = \omega_0)$ calculated at each surface S_j is represented in a speed color-coded 3D volume.

1. Supplementary Methods

1.1 Theoretical derivations on reverberant shear wave fields

As stated in the manuscript, a reverberant field can be understood as the superposition of plane shear waves traveling in a random direction[1]. Shear waves are transversal body-type waves; therefore, the particle velocity produced by these perturbations is perpendicular to the direction of propagation. For a given 3D point $\boldsymbol{\varepsilon}$ in the Cartesian system, three orthogonal vectors in the spherical coordinate system are found: $\hat{\mathbf{r}}$, $\hat{\boldsymbol{\theta}}$, and $\hat{\boldsymbol{\varphi}}$ (Figure 1a in the main manuscript). Then, the corresponding particle velocity vector field $\mathbf{V}(\boldsymbol{\varepsilon}, t)$, at position $\boldsymbol{\varepsilon}$ and time t, in a reverberant chamber produced by plane waves propagating with a wave number k and radial frequency ω_0 is modeled as

$$\mathbf{V}(\boldsymbol{\varepsilon}, t) = \sum_{q,l} \hat{\mathbf{n}}_{ql} v_{ql}\, \mathrm{e}^{\mathrm{i}(k\hat{\mathbf{n}}_q \cdot \boldsymbol{\varepsilon} - \omega_0 t)} \tag{1}$$

where the index q represents a realization of the random unit vector $\hat{\mathbf{n}}_q$ describing the direction of wave propagation, and the index l represents a realization of the random unit vector $\hat{\mathbf{n}}_{ql}$ describing a direction of particle velocity parallel to the disk formed by the basis vectors $\hat{\boldsymbol{\theta}}$ and $\hat{\boldsymbol{\varphi}}$ defined within a realization of q. Then, $\hat{\mathbf{n}}_q \cdot \hat{\mathbf{n}}_{ql} = 0$. Finally, v_{ql} is an independent, identically distributed random variable describing the magnitude of particle velocity within a realization of q. The summation over q is understood to be taken over the 4π solid angle, and the summation over l is taken over a 2π angle within the disk.

Typically, optical coherence tomography (OCT) systems measure particle velocity or displacement along one single axis that we call the sensor axis. For simplicity in the mathematical derivations, we choose the *x*-axis $\hat{\mathbf{e}}_x$ to be the sensor axis. Then, $V_x(\boldsymbol{\varepsilon}, t) = \mathbf{V}(\boldsymbol{\varepsilon}, t) \cdot \hat{\mathbf{e}}_x$ describes the particle velocity scalar field along $\hat{\mathbf{e}}_x$, and

$$V_x(\boldsymbol{\varepsilon}, t) = \mathbf{V}(\boldsymbol{\varepsilon}, t) \cdot \hat{\mathbf{e}}_x = \sum_{q,l} n_{ql_x} v_{ql}\, \mathrm{e}^{\mathrm{i}(k\hat{\mathbf{n}}_q \cdot \boldsymbol{\varepsilon} - \omega_0 t)} \tag{2}$$

where $n_{ql_x} = \hat{\mathbf{n}}_{ql} \cdot \hat{\mathbf{e}}_x$ is a scalar random variable. Then, using the spherical coordinate system (see definition of angles in Figure 1a in the main manuscript), we can represent $\hat{\mathbf{n}}_{ql} = \cos(\alpha)\, \hat{\boldsymbol{\varphi}} + \sin(\alpha)\hat{\boldsymbol{\theta}}$, where $\hat{\boldsymbol{\theta}} = \cos(\theta)\cos(\varphi)\hat{\mathbf{e}}_x + \cos(\theta)\sin(\varphi)\hat{\mathbf{e}}_y - \sin(\theta)\,\hat{\mathbf{e}}_z$, and $\hat{\boldsymbol{\varphi}} = -\sin(\varphi)\hat{\mathbf{e}}_x + \cos(\varphi)\hat{\mathbf{e}}_y + 0\,\hat{\mathbf{e}}_z$. Here, α is defined as the angle between $\hat{\mathbf{n}}_{ql}$ and $\hat{\boldsymbol{\varphi}}$, defining a realization of $\hat{\mathbf{n}}_{ql}$ within the disk formed by the basis vectors $\hat{\boldsymbol{\theta}}$ and $\hat{\boldsymbol{\varphi}}$. Therefore, we find that

$$n_{ql_x} = \hat{\mathbf{n}}_{ql} \cdot \hat{\mathbf{e}}_x = \sin(\alpha)\cos(\theta)\cos(\varphi) - \cos(\alpha)\sin(\varphi). \tag{3}$$

By taking the auto-correlation function of Equation 2, $B_{V_xV_x}$, in space and time, we obtain:

$$B_{V_xV_x}(\Delta\boldsymbol{\varepsilon},\Delta t) = E\{V_x(\boldsymbol{\varepsilon},t)V_x{}^*(\boldsymbol{\varepsilon}+\Delta\boldsymbol{\varepsilon},t+\Delta t)\} \tag{4a}$$

$$B_{V_xV_x}(\Delta\boldsymbol{\varepsilon},\Delta t) = E\left\{\left(\sum_{q,l} n_{ql_x} v_{ql}\, \mathrm{e}^{\mathrm{i}(k\hat{\mathbf{n}}_q\cdot\boldsymbol{\varepsilon}-\omega_0 t)}\right) \times \left(\sum_{q',l'} n_{q'l'_x} v_{q'l'}\, \mathrm{e}^{-\mathrm{i}(k\hat{\mathbf{n}}_{q'}\cdot(\boldsymbol{\varepsilon}+\Delta\boldsymbol{\varepsilon})-\omega_0(t+\Delta t))}\right)\right\} \tag{4b}$$

where $E\{\cdot\}$ represents an ensemble average and the asterisk represents conjugation. The product of the two series will include vanishing cross terms of the form:

$$E\left\{n_{ql_x} v_{ql}\, n_{q'l'_x}\, v_{q'l'}\, \mathrm{e}^{[\cdots]}\right\} = 0 \tag{5}$$

Since n_{ql_x} and v_{ql} are independent, and realizations of v_{ql} are uncorrelated, Equation 4b becomes:

$$B_{V_xV_x}(\Delta\boldsymbol{\varepsilon},\Delta t) = E\left\{\sum_{q,l} n_{ql_x}^2 v_{ql}^2\, \mathrm{e}^{\mathrm{i}(\omega_0\Delta t-k\hat{\mathbf{n}}_q\cdot\Delta\boldsymbol{\varepsilon})}\right\} \tag{6a}$$

$$B_{V_xV_x}(\Delta\boldsymbol{\varepsilon},\Delta t) = \beta E\left\{\sum_{q,l} n_{ql_x}^2\, \mathrm{e}^{\mathrm{i}(\omega_0\Delta t-k\hat{\mathbf{n}}_q\cdot\Delta\boldsymbol{\varepsilon})}\right\} \tag{6b}$$

where $\beta = \langle v_{ql}^2\rangle_{ql}$ is the expected value of v_{ql}^2 over both q and l realizations extracted from the curly braces since v_{ql} and $\{n_{ql_x}, \hat{\mathbf{n}}_q\}$ are independent random variables. In an ideal diffuse field, the ensemble or spatial averaging will assign equal weighting to all directions $\hat{\mathbf{n}}_q$ of incident shear waves in the 4π solid angle, and, given a realization of $\hat{\mathbf{n}}_q$, equal weithting in the direction of particle velocity $\hat{\mathbf{n}}_{ql}$ in the 2π angle within the disk formed by the basis vectors $\hat{\boldsymbol{\theta}}$ and $\hat{\boldsymbol{\varphi}}$ (Figure 1a in the main manuscript). Then, the average of the summation over discrete directions of each incident wave becomes the average over all directions in the polar coordinate system[2]. By substituting Equation 3 into Equation 6b and averaging in the polar coordinate system we have:

$$B_{V_xV_x}(\Delta\boldsymbol{\varepsilon},\Delta t) = \frac{\beta}{4\pi}\int_{\varphi=0}^{2\pi}\int_{\theta=0}^{\pi}\frac{1}{2\pi}\int_{\alpha=0}^{2\pi}(\sin(\alpha)\cos(\theta)\cos(\varphi) - \cos(\alpha)\sin(\varphi))^2\, \mathrm{e}^{\mathrm{i}(\omega_0\Delta t-k\hat{\mathbf{n}}_q\cdot\Delta\boldsymbol{\varepsilon})}\,\partial\alpha\,\partial\Omega \tag{7}$$

where $\partial\Omega = \sin(\theta)\,\partial\theta\,\partial\varphi$. For solving Equation 7, two cases are considered: the auto-correlation direction (1) perpendicular, and (2) parallel to the sensor axis. For case 1, we first consider $\Delta\boldsymbol{\varepsilon} = \Delta\boldsymbol{\varepsilon}_z$ along the *z*-axis. Then, the exponential term $k\hat{\mathbf{n}}_q \cdot \Delta\boldsymbol{\varepsilon}$ in Equation 7 becomes:

$$k\hat{\mathbf{n}}_q \cdot \Delta\boldsymbol{\varepsilon}_z = k\Delta\varepsilon_z \cos(\theta) \tag{8}$$

where $\Delta\varepsilon_z \cos(\theta)$ is the projection of $\hat{\mathbf{n}}_q$ in $\Delta\boldsymbol{\varepsilon}_z$ using spherical coordinates. Next, solving the triple integral in Equation 7 gives:

$$B_{V_x V_x}(\Delta\varepsilon_z, \Delta t) = \frac{\beta}{2} \mathrm{e}^{\mathrm{i}\omega_0 \Delta t} \cdot \left[j_0(k\Delta\varepsilon_z) - \frac{j_1(k\Delta\varepsilon_z)}{k\Delta\varepsilon_z} \right] \tag{9}$$

where j_0 and j_1 are spherical Bessel functions of the first kind of zero and first order, respectively. It is observed that the spatial and temporal component of $B_{V_x V_x}(\Delta\varepsilon_z, \Delta t)$ are separable and can be redefined as $B_{V_x V_x}(\Delta\varepsilon_z) B_{V_x V_x}(\Delta t)$. An identical result (when replacing the subscripts '*z*' in Equation 9 with '*y*') can be found when the *y*-axis is selected as the auto-correlation axis, $\Delta\boldsymbol{\varepsilon} = \Delta\boldsymbol{\varepsilon}_y$, since it is perpendicular to the sensor axis $\hat{\mathbf{e}}_x$. Finally, in order to be consistent with the main manuscript, we can redefine the sensor axis to be $\hat{\mathbf{e}}_z$ and the correlation axis to be $\hat{\mathbf{e}}_x$ or $\hat{\mathbf{e}}_y$ and change the subscripts accordingly since the ideal diffused field is isotropic. Then, accounting for the axis redefinition and neglecting the temporal component of Equation 9 we obtain Equation 2 of the main manuscript.

For case 2, we redefine the sensor axis to be $\hat{\mathbf{e}}_z$ and consider the auto-correlation direction $\Delta\boldsymbol{\varepsilon} = \Delta\boldsymbol{\varepsilon}_z$ along the *z*-axis (parallel to the sensor axis) for simplicity in the mathematical derivations. Then,

$$V_z(\boldsymbol{\varepsilon}, t) = \mathbf{V}(\boldsymbol{\varepsilon}, t) \cdot \hat{\mathbf{e}}_z = \sum_{q,l} n_{ql_z} v_{ql}\, \mathrm{e}^{\mathrm{i}(k\hat{\mathbf{n}}_q \cdot \boldsymbol{\varepsilon} - \omega_0 t)} \tag{10}$$

where $n_{ql_z} = \hat{\mathbf{n}}_{ql} \cdot \hat{\mathbf{e}}_z$ is the projection of $\hat{\mathbf{n}}_{ql}$ in the new sensor axis $\hat{\mathbf{e}}_z$. Following the same derivation flow as in Equation 3, we find that $n_{ql_z} = -\sin(\alpha)\sin(\theta)$. Then, taking the auto-correlation of Equation 10 along the *z*-axis and using $k\hat{\mathbf{n}}_q \cdot \Delta\boldsymbol{\varepsilon}_z = k\Delta\varepsilon_z \cos(\theta)$ in the exponential term, we have

$$B_{V_z V_z}(\Delta\varepsilon_z, \Delta t) = \frac{\beta}{4\pi} \int_{\varphi=0}^{2\pi} \int_{\theta=0}^{\pi} \frac{1}{2\pi} \int_{\alpha=0}^{2\pi} (-\sin(\alpha)\sin(\theta))^2\, \mathrm{e}^{\mathrm{i}(\omega_0 \Delta t - k\Delta\boldsymbol{\varepsilon}_z \cos(\theta))}\, \partial\alpha\, \partial\Omega \tag{11}$$

Solving the triple integral in Equation 11 gives:

$$B_{V_z\,V_z}(\Delta\varepsilon_z, \Delta t) = \frac{\beta}{2} \mathrm{e}^{\mathrm{i}\omega_0 \Delta t} \cdot \frac{j_1(k\Delta\varepsilon_z)}{k\Delta\varepsilon_z} \tag{12}$$

which is identical to Equation 3 of the main manuscript after neglecting the temporal component.

1.2 Spectral-domain phase-sensitive optical coherence tomography (PhS-OCT) system

The spectral-domain PhS-OCT used in the experiments was a custom-built system as shown in Supplementary Figure 3. The system was spectrometer-based with a light source coming from a superluminescent light emitting diode)EXS210045-01, EXALOS, Schlieren, Switzerland(with a central wavelength of 1307 nm and a full-width half-maximum)FWHM(spectral width of 100 nm. The average output power was 12 mW. The theoretical axial point-spread function)i.e. depth resolution limit(was computed to be approximately 8 μm.

The interferometer (see Supplementary Figure 3a) was designed based on a free-space Michelson interferometer configuration. The output from the light source was delivered to the interferometer through a single mode optical fiber. At the interferometer, the input light was collimated to a beam size of about 7 mm. The collimated light beam was split by a beam-splitter cube)BS015, Thorlabs, Newton, NJ, USA(, having 50:50 power split ratio. Half of the power of the light beam was delivered to a sample arm, consisting of dual-axes galvanometric mirrors)GVS012, Thorlabs, USA(, an objective lens)LSM05, Thorlabs, USA(, and a sample holder. The objective lens has a measured lateral resolution of about 15 μm, a working distance of about 90 mm, and a maximum scanning field of view of about 25 mm × 25 mm. The objective lens focused a light beam onto a sample and collected back-scattered light from the sample. The collected back-scattered light was, then, delivered back to the beam splitter to be combined with the reference light beam. An additional half-power of the light beam was propagated along the reference beam path which includes a dispersion compensator (LSM05DC, Thorlabs, USA) and a retro-reflector prism (PS975-C, Thorlabs, USA) mounted on a length-adjustable lens tube (SM1NR1, Thorlabs, USA). The reference light beam was reflected back and combined with the sample beam at the beam splitter. The combined light beam was coupled to another single mode fiber and delivered to a custom built spectrometer[3].

The spectrometer (see Supplementary Figure 3a) consisted of a reflective collimator, a reflective grating, a focusing mirror, and a line-array sensor. Light output at the other end of the fiber was placed at the focal point of a 90° off-axis parabolic (OAP) mirror with a reflective focal length of 101.6 mm (MPD249-M01, Thorlabs, USA) to produce a collimated beam of about 20 mm beam size. The collimated beam was directed to a reflective grating of 600 lines mm^{-1} (GR50-0613, Thorlabs, USA). A dispersed light beam was then focused by a 45° OAP mirror with a reflective focal length of 202.3 mm (MPD284-M01, Thorlabs, USA) to the line-array sensor. The line-array sensor was an InGaAs high speed line-scan camera, consisting of 2048 pixels of 10 μm pitch

(2048R, Sensor Unlimited, Princeton, NJ, USA)[3]. The spectral interference signal captured by the spectrometer was transferred to a computer for signal processing to obtain the depth profile signal. The signal processing consisted of interpolation of the captured spectrum to a linear wave number space, fast Fourier transformation, and logarithm mapping and display. The imaging depth, measured from a depth position of 10 dB amplitude drop, was approximately 5 mm. An axial resolution of the system was measured from the full width at FWHM of the depth profiles to be between 15-20 μm in air. The capturing of spectral interference signals was synchronized with the scanning of the light beam on the sample to produce 2D, 3D, or 4D OCT datasets. The Doppler phase shift detection scheme was implemented to monitor the propagation pattern of vibrational waves[4]. The data acquisition and OCT signal processing were implemented in the Labview program (Labview, National Instruments, Austin, TX, USA).

2. Supplementary References

1. Parker KJ, Maye BA. Partially coherent radiation from reverberant chambers. *The Journal of the Acoustical Society of America* **76**, 309-313 (1984).

2. Cook RK, Waterhouse RV, Berendt RD, Edelman S, Thompson MC. Measurement of Correlation Coefficients in Reverberant Sound Fields. *The Journal of the Acoustical Society of America* **27**, 1072-1077 (1955).

3. Pongchalee P, Meemon P, Widjaja J. Design of spectrometer-based frequency-domain optical coherence tomography at 1300 nm wavelength for skin diagnostics. In: *2017 10th Biomedical Engineering International Conference (BMEiCON)* (2017).

4. Pongchalee P, Palawong K, Meemon P. Implementation and characterization of phase-resolved Doppler optical coherence tomography method for flow velocity measurement. In: *International Conference on Experimental Mechanics 2013 and the Twelfth Asian Conference on Experimental Mechanics* SPIE (2014).
